# Information density threshold of urban road traffic signs based on visual comfort

**DOI:** 10.1016/j.heliyon.2024.e37080

**Published:** 2024-08-29

**Authors:** Hui Hu, Feng He, Yunwei Meng, Youzhang Yang, Binbin Li, Guangyan Qing

**Affiliations:** a Nanchang Urban Planning & Design Institute Group Co., Ltd., Nanchang, 330038, China; b Chongqing Key Laboratory of Intelligent Integrated and Multidimensional Transportation System, Chongqing Jiaotong University, Chongqing, 400074, China; c China Merchants Roadway Information Technology (Chongqing) Co., Ltd., Chongqing, 400067, China

**Keywords:** Urban road, Traffic sign, Real vehicle test, Visual comfort, Amount of information, Information density

## Abstract

The content and density of traffic signs directly affect the operation of urban road traffic and drivers. To overcome the limitations of quantitative research on the density threshold of traffic signs on urban roads, a real vehicle experiment was conducted to record the psychological characteristics of drivers. Four psychological parameters of drivers—pupil area, fixation intensity, heart rate change rate, and heart rate variability—were explored. Subsequently, principal component analysis was used to present a new index, S, divided into 5 grade scales, to represent the driving visual comfort level. The information entropy theory was applied to quantify the amount of information on road traffic signs that are included in driving tests, and a regression relationship between the traffic sign information and comfort index S was established. The visual psychological load thresholds for different comfort levels were −2.289≤S < −1.526 for very comfortable, 1.526≤S < −0.763 for relatively comfortable, −0.763≤S ≤ 0.763 for comfortable, 0.763<S ≤ 1.526 for uncomfortable, and 1.526<S ≤ 2.289 for very uncomfortable. To maintain the visual comfort of drivers, the information density of traffic signs should be less than 0.373 bits/m and the maximum information density of traffic signs should not exceed 0.507 bits/m. This conclusion provides a reference for the rational layout of traffic signs on urban roads.

## 1 Introduction

Urban roads play an important role in urban transportation. The driving environment on urban roads has distinct characteristics. During the construction and operation of urban roads, builders have an insufficient understanding of traffic signs and focus only on the road layout, which leads to traffic sign information overload along the road and increases the driving burden [1]. During driving, drivers collect and process road information primarily through visual channels, such as traffic signs, to maintain suitable driving conditions [2]. Road traffic signs that contain too little or too much information increase the psychological load and driving fatigue of drivers.

Because drivers experience certain psychological loads during the driving process, the working state of a driver can be represented by the driving load. This ensures safe driving only within the proper load range. The driving load and visual comfort index are closely related [3,4]; that is, a larger driving visual psychological load level corresponds to a lower driving comfort and vice versa. On urban roads, drivers frequently operate a car to keep it within a lane, turn, or change lanes, and these maneuvers are closely related to driving safety.

Many methods are used to study driving load and comfort. These can be divided into subjective index, driving operation, and psychological index assessment methods [5]. The psychological index assessment method can collect the real-time status of drivers, is more reliable that other methods, and has been widely applied [[6], [7], [8]].

When driving on urban roads, drivers often observe traffic signs, particularly when turning or changing lanes. Traffic signs can provide drivers with guidance regarding correct travel directions. When traffic signs are set up well, drivers feel safe and comfortable while driving; otherwise, driving may feel laborious.

An effective way of improving driver safety and comfort is to study the reasonable density range of road traffic signs from the perspective of driver visual comfort. Taking an urban road as the research scene, this study collected the psychological parameters of drivers using experimental methods, and their validity was verified. A driving visual psychological load model and evaluation method of visual comfort were proposed. A method for quantifying road traffic sign information was proposed, and the correlation between the information on road traffic signs and the visual comfort of drivers was studied.

The purpose of this study is to explore the visual psychological load model of urban roads and the influence mechanism of traffic sign information on the visual comfort of drivers to provide a reference for the rational layout of traffic signs. By employing principal component analysis (PCA), this study conducted multidimensional fusion research on driving comfort using indicators such as pupil area, fixation intensity, heart rate change rate, and heart rate variability. The information entropy theory was used to quantify the information content of the indicators.

## 2 Literature review

When driving on urban roads, drivers encounter many scenes outside the car, including traffic signs. Drivers continuously receive information provided by traffic signs, particularly when the driving environment is complex such as when passing through intersections or when there are many vehicles. During this process, drivers experience a certain psychological load that affects their safety and comfort. Therefore, determining a reasonable amount of information and layout density for traffic signs have become the focus of experts and scholars. Notably, the psychological load examined in this study was based on the cognitive load of traffic signs within a limited period and did not include driving fatigue.

During the tests, various psychological indicators of the drivers were gathered to assess the load and comfort experienced by the drivers while driving. These indicators were obtained through real driving tests and driving simulator tests and served as the primary basis for evaluating driving comfort. Scholars have conducted in-depth investigations into the fluctuation patterns of these indicators under different influencing factors and made significant research findings [9,10].

Comfort, experienced during driving or other activities, is subjective. Some studies have suggested that comfort is a psychological sensation that arises in a particular environment [11]. Other studies have posited that comfort is a bodily response triggered by the surrounding environment [12,13]. The visual comfort of drivers refers to the level of psychological satisfaction derived from observing the elements outside the vehicle, including the road landscape, terrain conditions, road types, and information provided by traffic signs. Scholars believe that when a driver is overloaded, they will experience rapidly changing pupil sizes, an increased heart rate, and an extended fixation time, indicating a decrease in driving comfort [14,15]. The mechanisms of mutual influence between the road environment and visual comfort have become a hot topic for many scholars.

Lowden [16] used a driving simulator to study the changes in the EEG (i.e. Electroencephalograms) and salivary cortisol at night under different psychological load states. The results showed that the EEG frequency and salivary cortisol levels were consistent with changes in the psychological load and driving comfort.

Tasaki [17] found that the ECG (i.e. Electrocardiogram) data of drivers could be used as a direct evaluation and judgment indicator to infer their psychological load state.

Wang et al. [18] explored the visual comfort of drivers based on the mean road landscape color and driving time, and they concluded that the mean color value was negatively correlated with the heart rate of drivers, whereas driving time was positively correlated with the mean heart rate of drivers.

Hu et al. [19] proposed a method for dividing driving comfort into three levels based on an indicator of driving psychological load, namely, the growth values of heart rate, when studying the driving experience on highway longitudinal slope sections. They categorized driving comfort into three levels: “discomfort” when the driving load is high, “relatively comfortable” when the driving load is moderate, and “comfortable” when the driving load is low.

The impact of the amount of traffic sign information on driving comfort is mainly reflected in the visual recognition process of the drivers. The amount of information affects the workload, recognition time, and visual recognition distance of drivers. More traffic sign information implies more workload, longer recognition time, and longer visual recognition distances [20,21].

As a tool that guides driver behavior, road traffic signs directly affect the driving state of drivers. The visual workload, psychological pressure, and driving comfort of drivers are influenced by the amount of traffic sign information [22,23].

Quantifying the amount of traffic sign information and determining the threshold at which the amount of sign information affects driving comfort is currently the main research focus of researchers at present. Traffic sign component element calculation methods [24] and image element calculation methods [25] have been developed for these purposes.

The NCHRP (i.e. The National Cooperative Highway Research Program) employed field tests and laboratory experiments to investigate the impact of the quantity of information on drivers regarding highway directional signs. They developed regression models for groups of traffic signs and individual traffic signs in relation to the information quantity [26].

It is evident that there is a definitive relationship between the driving psychological load and driving comfort. Characterizing the driving status using driving comfort is more direct and easier to align with the driver's overall perception of the driving environment. Moreover, utilizing psychological indicators of drivers enables a more quantitative expression of the psychological load and driving comfort. Currently, research has predominantly examined driving comfort through the lens of the psychological parameters of drivers, with a relatively limited focus on the driving vision of traffic signs. Traffic signs remain a critical focal point for drivers during the driving process. Based on the existing research, there is a consensus on employing psychological indicators of drivers to gauge driving comfort, alongside established methods for analyzing the information content of traffic signs. However, there is a dearth of studies on visual comfort during driving and the establishment of reasonable thresholds for urban road traffic signs.

## 3 Real-vehicle test

### 3.1 Test design

In this study, a real-vehicle test method was used. Participants drove on urban roads with various traffic signs. During the driving process, special test instruments measured the eye movements, heart rate, and other psychological data of the driving subjects and then analyzed the data. Because this study involved the participation of subjects before the experiment, all subjects knew the purpose of the test and agreed to the use of the test data for research. During the test, all subjects received an economic remuneration of 150 yuan, and the study was approved by the ethics committee.

The real vehicle test was divided into three main steps: selecting the test road section, determining the subjects and test vehicle, and installing the test instrument in the vehicle. After completing the real vehicle test, the data were analyzed.

To obtain additional test data and facilitate the exploration of regularities, each subject drove back and forth twice along the test road section. The actual driving arrangement was as follows. The subjects were numbered 1 through 10. The tests were conducted based on the number of subjects. First, when subject 1 was testing, subject 2 remained in the vehicle. After arriving at the end of the test section, subject 1 rested for approximately 5 min; then, subject 2 drove to the beginning of the section, and so on, until the first round of the test was completed. Second, subjects 1 and 2 switched driving directions and completed the second round of the test. Third, the forward and reverse directions were repeated until the second round of testing was completed. Therefore, when all subjects were tested in two directions, the test was completed.

#### 3.1.1 Test road section

The test road section was an urban road in Nanchang City, Jiangxi Province, China. An on-site survey and data review were conducted, and it was found that the design and construction of this urban road fully complied with Chinese industry standards. Except for traffic signs, other factors such as landscape, road width, and traffic volume show little change, which meets the test requirements. Based on map measurements and relevant information, the length of the test roads was approximately 5 km, and the design speed was 60 km/h. Each lane is 3.5 m wide.

To ensure minimal interference from other vehicles and maintain the integrity of the test results, the traffic volume in the designated section was deliberately kept low, allowing subjects to navigate freely. This section is situated within Nanchang City on a typical two-way, four-lane urban road equipped with comprehensive traffic engineering infrastructure. The modest traffic flow on this road aligns with the requirements of this study.

#### 3.1.2 Subjects and test vehicle

During the test, it was important to minimize the influence of individual differences among drivers on the test results [[27], [28], [29]]. To fulfill this criterion, a specific number of subjects was required.

Currently, there is no consensus regarding the number of test drivers in the fields of road driver vision and psychological research. To ensure the accuracy of the test parameters, a certain number of participants must be guaranteed. The calculation formula of the minimum measured sample size under ideal conditions is $N={\left(\frac{\sigma K}{E}\right)}^{2}$, where N is the required sample size, *∂* is the standard deviation of the population, assuming that the standard deviation of the speed is 5–10 km/h, K is a constant, the statistic at the confidence level, when the confidence level is equal to 95 %, K = 1.96, and E is the allowable error. Assuming that the speed allowable error is 5 km/h, through calculation, when the minimum measured sample size of this test satisfies N ≥ 4, sufficient accuracy of the test data can be ensured.

To make the test results reliable, considering the on-site conditions of the test road section, ease of operation of the vehicle, impact of the subjective initiative of drivers, and individual differences, 10 drivers were selected for this test, including six males and four female drivers with normal or corrected-to-normal vision. The age of the drivers selected for the test was 20–50 years and were free from any negative driving habits. Their demographics and traits were aligned with those typical of their region. None of the drivers were familiar with the specific road sections tested. Given the inherent risks of driving, these individuals held valid driving licenses and had at least 3 years of driving experience. Moreover, they were instructed to be in a good physical condition before the test, maintain a normal diet, get adequate rest, and ensure that their living conditions were conducive to safe driving.

A Buick commercial vehicle was used with the subject in the driver's seat. A test participant in the back seat was responsible for checking the test instruments at any given time. The test instruments were installed in the test vehicle.

#### 3.1.3 Test instruments

In the test, a Dikablis glasses-type tracker was used to collect eye movement data such as the driver's pupil area and fixation behavior. A varioport psychological recorder was used to collect ECG data from the driver. The eye tracker was equipped with cameras that recorded the environment inside and outside the car at a sampling frequency of 50 Hz. The sampling frequency of the physiological recorder was 100 Hz.

### 3.2 Test procedure

At the start of the test, the drivers gathered at the starting point of the road section. The physical condition of the drivers was confirmed to be normal to ensure that they were in good health and energetic. The test equipment was then adjusted uniformly, and the drivers were equipped with eye trackers and psychological recorders. The test commenced once the checks were completed.

**Fig. 1:**
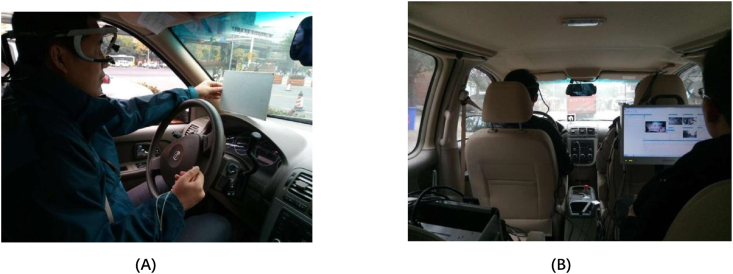
Test driver (A) and test participant (B) during the test process.

The vehicle traveled from the beginning to the end of the road section, with a test participant in the back seat responsible for operating the test equipment and recording the driving time and corresponding visual and psychological indicators, as illustrated in Fig. 1. Upon reaching the end of the test section, the driver took a 5-min break before completing the original route return test to complete the driving cycle. Subsequently, the next group of drivers underwent the same experimental process until all drivers completed the test.

### 3.3 Multi-characteristic parameter research

According to the relevant research [[30], [31], [32], [33]], the indicators used to measure driving comfort vary. The influence of the age and sex of the subjects was not considered during data analysis. Four psychological indices—change rate of the pupil area, fixation intensity, change rate of the heartbeat, and heart rate variability—were selected for a comprehensive analysis based on the objectives of the experiment and the data collected.

According to the sampling frequency of the test instrument, each of the ten drivers conducted two round-trip tests on the same road section. Approximately 600,000 eye movement data points and 1.2 million heart rate data points were collected. A clustering method was used to test the data validity. Since the driver's psychological reaction during the test was relatively stable, data exceeding the 20 % limit of the average value were eliminated, and the driver's data of the starting and ending road section were also removed, leaving 428,040 eye movement data points and 882,720 heart rate data points for the study. The amount of data was sufficient for the study objective. The subsequent analysis was based on valid data, and the correlation between the test data and traffic signs was studied.

#### 3.3.1 Change rate of pupil area

The rate of change of the pupil area [34] can be utilized as an indicator to evaluate psychological responses and driving comfort. The rate of change of the pupil area $R_{t}$ is defined as follows:

(1) $$R_{t}=\left|\frac{S_{2}-S_{1}}{S_{1}}\right|\times 100\%\text{,}$$

where $S_{2}$ is the pupil area during driving and $S_{1}$ is the pupil area before driving.

#### 3.3.2 Fixation intensity

The index of fixation intensity represents the driver's attention to traffic signs and their ability to process environment-related information at a specific speed. This is the product of the average fixation time for a single fixation point and the average number of fixations within a unit time of the driving section [35], which can be expressed as

(2) $$a=T_{a}N_{a}\text{,}$$

where *a* is the driver's fixation intensity, $T_{a}$ is the average fixation time of a single fixation point (s), and $N_{a}$ is the average number of fixations per unit time (times/s).

#### 3.3.3 Change rate of heartbeat

The change rate of the heartbeat can reflect the driver's feelings of impatience [36]. At the same time, when the change rate of the heartbeat exceeds 20 %, drivers experience an increase in psychological tension, and driving comfort is reduced accordingly. A quantitative approach was used to accurately analyze driver heartbeat changes at certain times or periods. The calculation of the change rate of the heartbeat *Ni* is as follows:

(3) $$Ni=\left|\frac{HR_{i}-HR_{0}}{HR_{0}}\right|\times 100\%\text{,}$$

where $HR_{i}$ is the heartbeat and $HR_{0}$ is the average heartbeat per unit time.

#### 3.3.4 Heart rate variability

Heart rate variability, namely, the standard deviation of the heartbeat interval, is a commonly used temporal analysis indicator [37]. The standard deviation of NN intervals, SDNN, was chosen as the main indicator to characterize the drivers' heart rate variability [38,39]. The SDNN of the driver's heart rate variability was calculated every 30 s. The formula for calculating SDNN is

(4) $$SDNN=\sqrt{\frac{1}{N-1}\sum_{i=1}^{N}{\left(r_{i}-\bar{r}\right)}^{2}}\text{,}$$

where N is the total number of R waves in this period, *r*_*i*_ is the *ith* NN interval size, and $\bar{r}$ is the average NN interval in the time range.

### 3.4 Multi-feature fusion of driving visual comfort

#### 3.4.1 Correlation analysis of characteristic parameters

The K-S normal distribution test was performed on the rate of change of the pupil area, fixation intensity, rate of change of the heartbeat, and SDNN. The test results are listed in Table 1.

**Table 1:** Test results of normality hypothesis for each data.

Null hypothesis	Significance level	Decision
The distribution of the change rate of the pupil area was found to be normal, with a mean of 8.11 and a standard deviation of 1.92.	0.075	Keep the null hypothesis
The distribution of the fixation intensity was found to be normal, with a mean of 6.59 and a standard deviation of 0.43.	0.061	Keep the null hypothesis
The distribution of the change rate of the heartbeat was found to be normal, with a mean of 79.916 and a standard deviation of 7.745.	0.099	Keep the null hypothesis
The distribution of the heart rate variability was found to be normal, with a mean of 65.16 and a standard deviation of 4.32.	0.089	Keep the null hypothesis

According to the results of the normal distribution test, the significance levels of the normal test for the change rate of the pupil area, fixation intensity, change rate of the heartbeat, and heart rate variability were all higher than 0.05, which proved that the parameters were normally distributed and could be used for data analysis.

An analysis was conducted to explore the correlation between the driver visual comfort indices. The results are summarized in Table 2.

**Table 2:** Correlation analysis of visual comfort index.

Visual comfort index	Pearson analysis	Change rate of pupil area	Fixation intensity	Change rate of heartbeat	Heart rate variability
Change rate of pupil area	Pearson correlation	1.000			
	Significance (bilateral)				
Fixation intensity	Pearson correlation	0.646	1.000		
	Significance (bilateral)	0.000			
Change rate of heartbeat	Pearson correlation	0.395	0.337	1.000	
	Significance (bilateral)	0.000	0.000		
Heart rate variability	Pearson correlation	−0.296	−0.241	−0.547	1.000
	Significance (bilateral)	0.002	0.013	0.000	

As shown in Table 2, the change rate of the pupil area, fixation intensity, change rate of the heartbeat, and heart rate variability were significantly correlated with each other, which can be further integrated and extracted to facilitate subsequent analyses.

#### 3.4.2 Calculation model of visual load

PCA is a data processing method for extracting common elements from sample variables and can integrate multiple indicators into a single or few indicators. The Kaiser-Meyer-Olkin (KMO) and Bartlett tests of sphericity were employed to confirm the suitability of the samples and test the correlation of the samples [40].

When the KMO test value is greater than 0.5 or the P value of the Bartlett ’s test of sphericity is less than 0.05, the test passes, and each characteristic parameter can be considered suitable for factor analysis.

Bartlett's test of sphericity and the KMO test were performed on the change rate of the pupil area, fixation intensity, change rate of the heartbeat, and heart rate variability, and the test results are shown in Table 3.

**Table 3:** KMO and Bartlett tests.

Sampling enough for Kaiser-Meyer-Olkin metric.		0.642
Bartlett test of sphericity	Approximate chi-square	111.129
	*d*_*f*_	6
	*Sig.*	0.000

From Table 3, the KMO test value is 0.642, and Bartlett's test of sphericity *sig.* value is 0.000. Each characteristic parameter was considered suitable for factor analysis.

The Z-A score normalization method was adopted to standardize the test data and determine the common factors, and the variance interpretation rate of each factor was obtained, as shown in Table 4.

**Table 4:** Interpret total variances.

Index	Initial eigenvalue			Extract the sum of squares		
	Total	Variance (%)	Accumulation (%)	Total	Variance (%)	Accumulation (%)
Change rate of pupil area	2.237	55.930	55.930	2.237	55.930	55.930
Fixation intensity	0.971	24.281	80.211	0.971	24.281	80.211
Change rate of heartbeat	0.442	11.042	91.253			
Heart rate variability	0.350	8.474	100.000			

The Z-score standardization method was used to standardize the experimental data, and common factors were determined to obtain the variance explanation rate for each factor, as shown in Table 4.

The selection of the common indicators was based on the analysis of variables obtained through gravel testing. By extracting the two common factors, a large portion of the original variable information can be effectively represented. The factor-loading matrices are listed in Table 5.

**Table 5:** Factor loading matrix.

Factors	Loading score	
	1	2
Change rate of pupil area	0.798	0.42
Fixation intensity	0.755	0.512
Change rate of heartbeat	0.759	−0.421
Heart rate variability	−0.674	0.597

The factor loading matrix is the score of the two common factors in the psychological parameters of drivers; that is, to express the influence of each characteristic parameter on the two common factors, the linear relationship between each characteristic parameter and the common factor can be obtained through the component matrix, as follows:

(5) $${\tilde{X}}_{1}=0.798F_{1}+0.420F_{2}+\varepsilon_{i}\text{,}$$

where ${\tilde{X}}_{1}$ is the change rate of the pupil area after standardization, $F_{1}$ is common factor 1, and $F_{2}$ is common factor 2, which is a special factor ${\tilde{X}}_{1}$.

Each factor was rotated to better explain the extracted factors. Using the orthogonal rotation method of maximum variance, all factors remain in the orthogonal state, and the variance difference of all factors reaches a maximum; that is, the sum of squares of the relative load reaches a maximum. The scoring matrix after factor rotation is presented in Table 6.

**Table 6:** Component matrix after factor rotation.

Index	Ingredient	
	1	2
Change rate of pupil area	0.872	0.230
Fixation intensity	0.903	0.133
Change rate of heartbeat	0.275	0.823
Heart rate variability	−0.094	−0.895

The orthogonal rotation method can classify the change rate of the pupil area and fixation intensity into one category, named the “eye movement load factor,” which represents the psychological reaction of drivers in eye movement. The change rate of the 2 heartbeat and heart rate variability are classified as “heartbeat load factor,” which represents the psychological response of the driver in terms of heartbeat.

The linear relationship between each characteristic parameter and the common factor can be obtained using the component matrix, and the common factor can also be expressed by each characteristic parameter. The scoring factor is a linear function. The regression estimation method was used to determine the score coefficient matrix of the eye movement and heartbeat load factors, as shown in Table 7.

**Table 7:** Score coefficient matrix of each component.

Index	Ingredient	
	1	2
Change rate of pupil area	0.555	−0.078
Fixation intensity	0.605	−0.161
Change rate of heartbeat	−0.043	0.549
Heart rate variability	0.193	−0.656

In Table 7, each value is the extracted score coefficient of the eye movement and heartbeat load factors of the drivers. Therefore, the expression of the score coefficients of the eye movement and heartbeat load factors can be obtained using each characteristic parameter, as follows:

(6) $$\begin{array}{c} F_{1}=0.555{\tilde{x}}_{1}+0.605{\tilde{x}}_{2}‐0.043{\tilde{x}}_{3}+0.193{\tilde{x}}_{4}\text{,}\\ F_{2}=‐0.078{\tilde{x}}_{1}‐0.161{\tilde{x}}_{2}+0.549{\tilde{x}}_{3}‐0.656{\tilde{x}}_{4}\text{,} \end{array}$$

where $F_{1}$ is the eye movement load factor, $F_{2}$ is the heartbeat load factor, ${\tilde{x}}_{1}$ is an indicator of the change rate of the pupil area after standardization, ${\tilde{x}}_{2}$ is an indicator of the fixation intensity after standardization, ${\tilde{x}}_{3}$ is an indicator of the change rate of the heartbeat after standardization, and ${\tilde{x}}_{4}$ is an SDNN indicator of heart rate variability after standardization.

The calculation model of the psychological load of the drivers can be obtained using the factor score coefficient and variance contribution through the above analysis, as follows:

(7) $$S=\frac{55.93F_{1}+24.28F_{2}}{80.21}=0.697F_{1}+0.303F_{2}\text{,}$$

where S is the level of driving visual psychological load. This equation combines the commonalities and advantages of the eye movements and heartbeat characteristics of the drivers, and it can better reflect the actual psychological status of drivers.

When analyzing the data distribution of the visual psychological load of the drivers, the level of visual psychological load was obtained from each standardized influencing factor. After the data analysis, each factor was consistent with a normal distribution, and the visual psychological load level was consistent with a normal distribution. The results of the normality tests are presented in Table 8.

**Table 8:** Visual psychological load normal hypothesis test.

Null hypothesis	Test method	Significance level	Decision
The distribution of visual psychological load is normal distribution	Kolmogorov-Smirnov test	0.156	Keep the null hypothesis

#### 3.4.3 Classification of visual comfort level

**Fig. 2:**
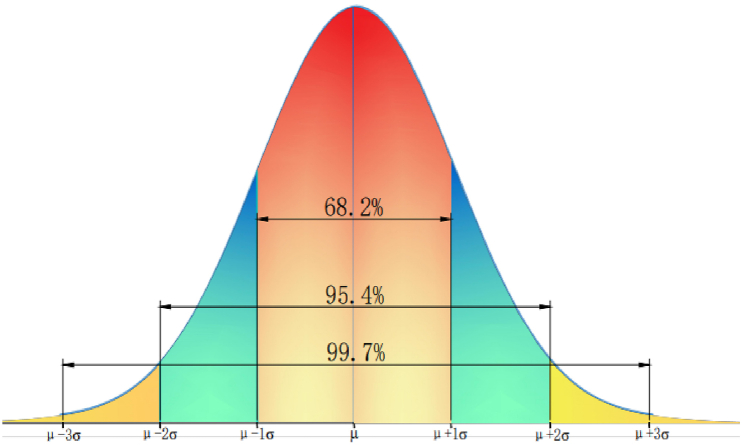
Probability distribution curve of normal distribution.

By analyzing the collected visual psychological data, it was found that the significance value of the normality test result was 0.156, which was greater than 0.05, indicating that the visual psychological load level meets the normality test. For a set of randomly distributed data, the distribution was mostly concentrated around the mean, particularly for data that conformed to the normality. Most observations were clustered around the central peak (mean) with the highest probability of occurrence, as shown in Fig. 2. As the data deviate from the central peak in both directions, the data appearing in the tail of the curve are increasingly less likely, with a probability of almost 1. This is within three standard deviations of the mathematically expected range for a set of normal random variables. The probability that the numerical value is distributed in (μ-σ, μ+σ) and (μ-2σ, μ+2σ) is 0.6826 and 0.9544, respectively. The probability that the numerical value is distributed in the range of (μ-3σ, μ+3σ) is 0.9974, which is called the “3σ" principle of normal distribution, where σ is the standard deviation, μ is the mean, and *x* = μ is the axis of symmetry of the image.

Therefore, according to the mathematical principle, taking (μ-3σ, μ+3σ) as the actual possible value range of the visual psychological load of the drivers, the visual comfort of the drivers was divided by three standard deviations of the probability distribution function of the visual psychological load level of the drivers. According to the different range of deviation, the visual comfort level was divided into x±σ,x±2σ,x±3σ as boundary points.

Table 9 presents the specific classification results for the visual comfort levels of the drivers.

**Table 9:** Classification of driving visual comfort.

Driving visual comfort level	Range of psychological load
Very comfortable	−2.289≤S < −1.526
More comfortable	−1.526≤S < −0.763
Comfortable	--0.763≤S ≤ 0.763
Uncomfortable	0.763<S ≤ 1.526
Very uncomfortable	1.526<S ≤ 2.289

## 4 Traffic sign information

### 4.1 Quantification method of urban road traffic sign information

The information volume calculation method for urban road traffic engineering facilities is based primarily on Shannon's information entropy theory, which calls the amount of information contained in the source information entropy and is used to describe the average size of event information. When it is necessary to measure and determine the amount of information that an unknown object has, the amount of information (unit: bits) is an effective measurement tool. When a random variable has a certain value, the logarithm of the reciprocal of its probability indicates the amount of information contained. The amount of information contained in an object is negatively correlated to its probability of occurrence. The greater the probability of occurrence, the smaller the uncertainty and the amount of information contained. Based on the information entropy theory and the basic components of traffic engineering, some scholars have constructed a calculation model for the basic information amount of traffic engineering facilities [34].

The mathematical expression for information $I_{i}$ is

(8) $$I_{i}=‐\log_{2}\;p\left(x_{i}\right)\text{,}$$

where *p* $\left(x_{i}\right)$ is the probability of the occurrence of event $x_{i}$.

Information entropy is the measurement of the information contained in a source, which is the expectation of the information contained in an event. Information entropy is the probability of the occurrence of each state multiplied by the sum of its information amount. According to Shannon's information theory, the calculation method for information in traffic engineering facilities is

(9) $$H\left(X\right)=-\sum_{i=1}^{n}p\left(x_{i}\right)\log_{2}\;p\left(x_{i}\right)\text{,}$$

where *X* is the traffic engineering facility event, *H(X)* is the amount of information (bits), *N* is the total number of possible states of event *X, Xi* is the *ith* state of the event, and *P(xi)* is the probability of event *x*_*i*_.

Traffic engineering facilities on urban roads include indication signs, warning signs, guiding signs, traffic markings, scenic spots, and guardrails with Chinese characters, English letters, Arabic numerals, colors, pointing arrows, shapes, and other forms [[41], [42], [43]]. When road conditions are good, traffic signs are the focus of driver attention.

It is assumed that events in each state will occur with the same probability; that is, each single element information of traffic signs will produce visual stimulation to drivers with the same probability. Therefore, the probability of the occurrence of elements of sign information of the counting type is *P(xi)* = *1/n* and that of the length type is *P(xi)* = *M/n*, where *M* is the length of an element's information type and *n* is the calculated length of all elements.

According to the equation for the information amount of traffic signs on an urban road, the calculation of a single information amount of counting-type elements can be simplified as follows:

(10) $$H\left(x_{i}\right)=\log_{2}\;n\text{.}$$

The amount of information for each element of the traffic signs can be obtained according to the above equation, and the calculation process is as follows.

(1) Chinese characters. According to the Table of Common Characters in Modern Chinese, there are approximately 3500 Chinese characters in daily use; therefore, each Chinese character contains *H(X*_*1*_*)* = log_2_3500 = 11.77 bits.
(2) English letters. There are 26 letters in English. Therefore, each letter contains *H(X*_*2*_*)* = log_2_52 = 5.70 bits.
(3) Arabic numerals. There are 10 Arabic numerals. Thus, each number contains *H(X*_*3*_*)* = log_2_10 = 3.32 bits.
(4) Color. Traffic signs primarily use six colors: black, white, green, blue, red, and yellow. There are also many brown tourist zone signs on urban roads; therefore, each color contains *H(x4)* = log_2_7 = 2.81 bits.
(5) Directional arrows. Approximately 40 types of directional arrows are used in the traffic signs on urban roads, and each arrow contains *H(x5)* = log_2_40 = 5.32 bits.
(6) Symbols. In contrast to ordinary roads, warning signs used on narrow roads and tunnels are rare on urban roads in Nanchang City. The actual local situation must be considered when calculating the amount of symbol information. According to the statistics, approximately 60 symbols indicate road conditions, attention, or vehicle types, and each symbol contains *H(x6)* = log_2_60 = 5.91 bits.
(7) Shape. There are six commonly used shapes of traffic signs on urban roads: triangle, square, circle, rectangle, octagon, and pentagonal, and each shape contains *H(x7)* = log_2_6 = 2.59 bits.

The calculation equation for the amount of information for each traffic sign can be obtained by integrating the sum of the information for all elements:

(11) $$H\left(X\right)=\sum \left(H\left(x_{i}\right),n\right)\text{,}$$

where *H(X)* is the amount of information on the traffic signs on urban roads (bits), *H(xi)* is the amount of information contained in a single element (bits), and *N* is the number of elements on the traffic sign.

The information transmitted by traffic signs is not evenly distributed along the urban section, so a comparative study can be conducted [44,45]. When driving on an urban road, drivers are affected by the information of traffic signs on both sides of the road; the information density of traffic signs is expressed as

(12) $$\rho \left(X\right)=\frac{H_{L}\left(x_{i}\right)+H_{S}\left(x_{i}\right)}{2S}\text{,}$$

where *ρ(X)* is the information density of urban road traffic signs, bits/km; *H*_*L*_*(x*_*i*_*)* and *H*_*S*_*(x*_*i*_*)* are the information amount of traffic signs on both sides of the road, bits; and *S* is the length of urban road, km.

For the driving test, the amount of information for some traffic signs are listed in Table 10. According to Equation (12) and Table 10, the information density in the test section can be calculated.

**Table 10:** Amount of information for some traffic signs.

Type of traffic signs	Each element in traffic sign							Amount of information(bits)
	Chinese characters	English letters	Arabic Number	Color	Arrows	Symbol	Shape	
	0	0	0	2	0	2	2	22.62
	0	0	0	2	0	2	2	22.62
	0	0	0	2	1	0	2	16.12
	0	0	2	3	0	0	2	20.25
	0	1	2	3	0	0	3	28.54
	0	1	2	3	0	1	3	34.45

### 4.2 Analysis of traffic sign information and visual comfort

The rate of change of the pupil area, fixation intensity, rate of change of the heartbeat, and heart rate variability were calculated [46]. A Pearson's correlation analysis was conducted, and the results are presented in Table 11.

**Table 11:** Parameters of traffic sign information density and visual comfort.

Correlation analysis with information density	Change rate of pupil area	Fixation intensity	Change rate of heartbeat	Heart rate variability
Pearson correlation	0.717	0763	0.631	−0.576
Significance (bilateral)	0.001	0.000	0.002	0.006

**Fig. 3:**
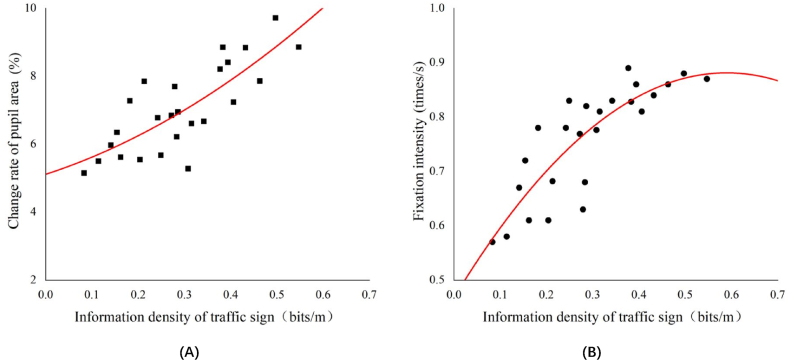
Relationship between change rate of pupil area (A), Fixation intensity (B) and information density of traffic sign respectively.

**Fig. 4:**
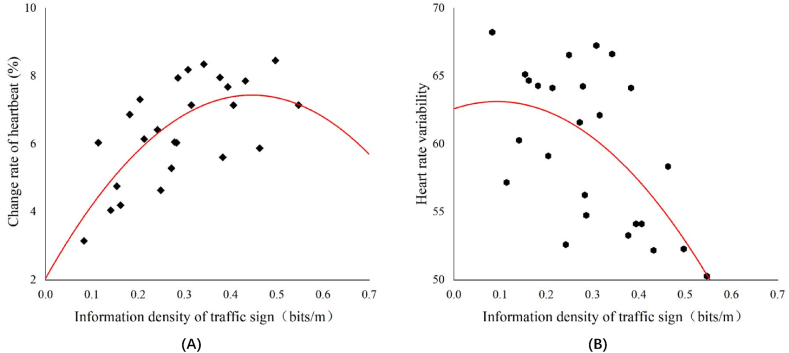
Relationship between change rate of heartbeat (A), heart rate variability (B) and information density of traffic sign respectively.

It can be seen from Table 11, the information density of traffic signs has a certain correlation with the eye movement and heart rate factors of drivers, which is strongly positively correlated with the change rate of the pupil area, strongly positively correlated with fixation intensity, moderately positively correlated with the change rate of the heartbeat, and moderately negatively correlated with the heart rate variability. The relationship between the rate of change in the pupil area, fixation intensity, and information density of traffic signs is shown in Fig. 3. The relationship between the rate of change in the heartbeat, heart rate variability, and information density is shown in Fig. 4.

As shown in Fig. 3, as the information density of the traffic signs increased, the change rate of the pupil area and fixation intensity showed an overall increasing trend. When driving on a road section with low information density, the change rate of the pupil area is small, and the fixation intensity is relatively low; therefore, the visual comfort of the driver is relatively high. As the information density of traffic signs increases, the rate of change in the pupil area and the fixation intensity of the driver also increases. Traffic signs place increasing requirements on the driver's ability to process information, causing pupil area fluctuations. This indicates that the psychological load of driving is constantly increasing. It is not until the information density of traffic signs reaches a certain value that the growth rate of the fixation intensity and the change rate of the pupil area begin to slow down.

As shown in Fig. 4, as the information density of the traffic sign increases, the rate of change of the heartbeat shows a trend of first increasing and then decreasing, whereas the heart rate variability shows a trend of first changing slowly and then significantly decreasing. This shows that excessive traffic-sign information produces a greater psychological load on the driver and increases driver stress. If the driver cannot fully accept the information on traffic signs, visual comfort and safety will be adversely affected.

### 4.3 Analysis of the correlation between information density of traffic signs and driving comfort

There is a certain degree of correspondence between the driving visual comfort and driving load [[47], [48], [49]]. The visual comfort level was obtained based on the driving load index. Based on this idea, a visual comfort regression model under different traffic sign information densities was established. The eye movement and heart rate load factors of the drivers were first calculated, and the corresponding visual load value was then calculated using the calculation equation of the comprehensive score. Thus, the correlation between the driving visual comfort level and the information density of traffic signs can be obtained, as shown in Table 12.

**Table 12:** Correlation between information density of traffic signs and visual comfort.

		Information density (bits/m)	Eye movement load	Heartbeat load	Visual psychological load
Information density (bits/m)	Pearson correlation	1			
	Significance (bilateral)				
Eye movement load	Pearson correlation	0.836	1		
	Significance (bilateral)	0.000			
Heartbeat load	Pearson correlation	0.579	0.336	1	
	Significance (bilateral)	0.006	0.100		
Visual psychological load	Pearson correlation	0.891	0.931	0.622	1
	Significance (bilateral)	0.000	0.000	0.001	

From Tables 11 and it is evident that the correlation between the eye movement load factor and information density of traffic signs is stronger than that between the heartbeat load factor and information density of traffic signs. This suggests a significant correlation between the visual psychological load of drivers and the information density of traffic signs.

**Fig. 5:**
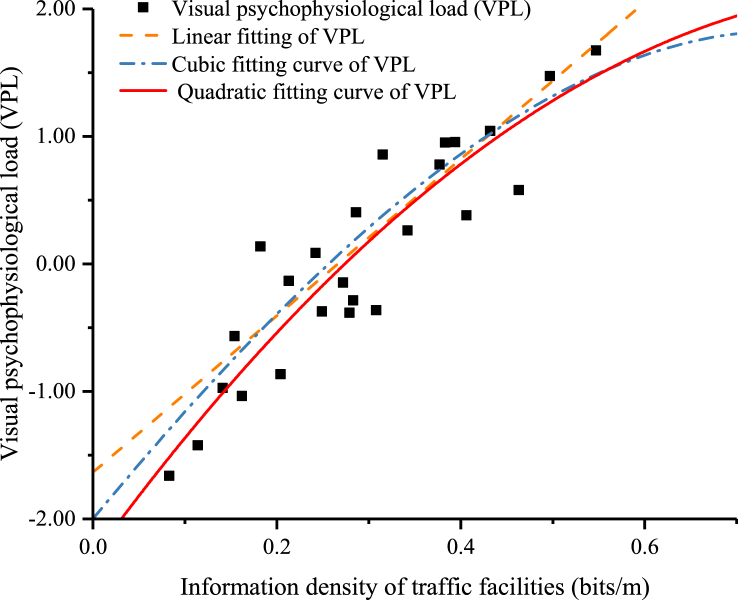
Fitting diagram of visual psychological load and information density of traffic signs.

Linear, quadratic, and cubic regression analyses were performed to investigate the specific relationship between the information density of traffic signs and the drivers' visual psychological load. The fitting of each regression function is shown in Fig. 5.

It is apparent from Fig. 5 that there is a discernible pattern in the distribution of the visual psychological load of the drivers across varying traffic sign information densities, indicating a quantifiable relationship. The results of the fitting parameters and F-tests for each regression model are presented in Table 13.

**Table 13:** Fitting parameters and F-test results.

Fitting equation form	The model parameters			F-test			
	R^2^	R squared after adjustment	Standard error	*d*_*f1*_	*d*_*f2*_	F	*Sig.*
Linear	0.764	0.753	0.410	1	23	76.70	0.00
Quadratic	0.774	0.754	0.410	2	22	33.78	0.00
Cubic	0.778	0.746	0.416	3	21	24.53	0.00

As shown in Table 13, all three models passed the F-test, showing that the models are valid. After adjusting the fitting models, the R-squared value from the quadratic curve was the largest (0.754) and its regression fitting effect was the best. Therefore, using a quadratic curve function to establish a quantitative relationship model between the information density of traffic signs on urban roads and the visual psychological load of drivers is the most accurate and effective approach. The regression model is given by

(13) $$S=-6.17x^{2}+11.11x-2.52\text{,}$$

where S is the visual psychological load and *x* is the information density of the traffic signs.

It can be seen from the regression model and quadratic curve fitting diagram that there is a negative correlation between the visual load and information density of traffic signs. Therefore, it can be concluded that when driving, the visual comfort of drivers is higher in sections with lower information density of traffic signs and lower in sections with higher information density.

### 4.4 Analysis of traffic sign information density based on visual comfort

Drawing on the correlation between the information density of traffic signs on urban roads and the visual load, a recommended range for setting the information density of traffic signs was proposed based on the visual load thresholds. In terms of driver visual comfort, the optimal range for the information density of traffic signs falls within the interval [0.021, 0.725]. When the information density exceeds this range, the visual load level S can be adjusted using the following equation:

(14) $$S=\left\{ \begin{array}{c} \begin{array}{cc} 2.289 & \left(0.725\leq x_{3}\right) \end{array}\\ \begin{array}{cc} -6.17x_{3}^{2}+11.11x_{3}-2.52 & \left(0.021.896\leq x_{3}\leq 0.725\right) \end{array}\\ \begin{array}{cc} -2.289 & \left(0\leq x_{3}\leq 0.021\right) \end{array} \end{array}\right.$$

The information density ranges of the traffic signs based on the different visual comfort levels are listed in Table 14.

**Table 14:** Range of information density of traffic sign on urban road for visual comfort.

Visual comfort level	S-threshold range of visual load	Information density of traffic signs
Very comfortable	−2.289≤*S* < −1.526	0≤x≤0.094
More comfortable	−1.526≤*S* < −0.763	0.094≤x≤0.175
Comfortable	--0.763≤*S* ≤ 0.763	0.175≤x≤0.373
Uncomfortable	0.763<*S* ≤ 1.526	0.373≤x≤0.507
Very uncomfortable	1.526<*S* ≤ 2.289	0.507≤x

The level of driving visual comfort is directly related to the psychological performance of drivers [50]. Based on the above analysis, a recommended setting range for the information density of urban road traffic signs was proposed. To ensure the visual comfort of drivers, the recommended value of the information density for urban road traffic signs should be less than 0.373 bits/m. This can keep the visual load of drivers low and maintain the visual comfort level above the “uncomfortable” level. Simultaneously, the maximum information density of urban road traffic signs should not exceed 0.507 bits/m.

## 5 Conclusion

To explore the relationship between the density of traffic signs on urban roads and driving comfort, we used a method that combines actual measurements and theoretical analysis to conduct a real vehicle test on a section of urban roads. We collected and analyzed the psychological parameters of drivers. A calculation model of the visual psychological load on urban roads was proposed, the relationship between the information content of urban road traffic signs and the visual comfort of drivers was discerned, and suggestions for setting up traffic signs were suggested. The following conclusions were drawn.

(1) The change rate of the pupil area, fixation intensity, change rate of the heartbeat, and heart rate variability are significantly correlated with each other, indicating that these four visual comfort indicators have certain correlations and shared common characteristics.
(2) A factor analysis conducted on these four indicators found the score coefficient of the eye movement load factor: $F_{1}=0.555{\tilde{x}}_{1}+0.605{\tilde{x}}_{2}-0.043{\tilde{x}}_{3}+0.193{\tilde{x}}_{4}$, the score coefficient of the heartbeat load factor: $F_{2}=-0.078{\tilde{x}}_{1}-0.161{\tilde{x}}_{2}+0.549{\tilde{x}}_{3}-0.656{\tilde{x}}_{4}$, and the calculation model of the visual psychological load of the driver: $S=0.697F_{1}+0.303F_{2}$.
(3) Information density *x* of traffic signs has a negative feedback effect on visual comfort and a strong positive correlation with the visual psychological load *S.* The relationship is $S=-6.17x^{2}+11.11x-2.52$. Based on studies regarding the visual psychological load levels, for drivers to maintain a safe state, the recommended information density of traffic signs on urban roads should be below 0.373 bits/m, with a maximum threshold of 0.507 bits/m for the information density of traffic signs.

## 6 Discussion

Linear road conditions and driver personal characteristics were not considered in this study. These factors may affect the driver's comfort threshold.

Meng [51] found that in the tunnel entrance section, the comfort of drivers is not only related to the setting of traffic signs but also to the environmental brightness and driving speed. Therefore, based on the comfort requirements of drivers, a corresponding brightness change rate curve was constructed.

Du [52] and his team conducted a study on the information density and driving comfort of traffic signs on grassland roads using a driving simulator. They believed that when the traffic sign density is not greater than 30 bits/km, driver comfort can be guaranteed. In this study, the traffic sign density corresponding to the given comfortable state is 0.094 bits/m, which is 94 bit/km, and there is a certain difference from the value in Ref. [52]. We believe that the formation of such differences is mainly due to two factors: different experimental methods and different research objects. This study used real vehicle tests to study the density of traffic signs on urban roads. Compared with grassland roads, traffic signs are more densely set on urban roads, and drivers have different degrees of adaptation to this driving environment compared with grassland roads. Generally, on urban roads, drivers can tolerate more traffic signs and the actual traffic conditions require more traffic signs.

Liu [21] recorded the experiences of eight adult passengers through actual vehicle testing to evaluate driving comfort on urban road sections. Their experiment revealed that excessive acceleration causes discomfort to passengers. Although this study did not directly affect the volume of traffic signs, driving speed on road sections was easily affected by these signs. This indirectly proves the relationship between traffic sign settings and driving comfort.

Differences in gender, age, and driving experience can lead to differences in the visual recognition distances for drivers in relation to road signs [53]. We believe that inappropriate linear road conditions may cause drivers to focus more on road condition information collection and vehicle operation control. This means that the time required for drivers to recognize the sign information is reduced, and there may be different corresponding relationships between the same amount of sign information and driver comfort.

Additionally, because of the different road parameters of urban roads and expressways, the traffic volume and composition of roads should be considered to explore whether these factors affect the threshold of sign information content.

In this study, a real vehicle test was used to conduct a quantitative analysis on the reasonable density of traffic signs on urban roads. During the test, the amount of data obtained from the real experiments was large, which was an advantage. However, this method is expensive, requires professional testing equipment, and requires a group of subject drivers. This is an obvious shortcoming of the research method. In subsequent research, a driving simulator research method can be used; however, the driving scenarios must be sufficient. It is recommended that real driving scenes be produced as video files for use by drivers in driving simulators. Further research should focus on the visibility of traffic signs in urban road sections with different traffic volumes under different weather conditions.

In addition, the effective recognition of traffic signs is an important technology in current research on autonomous driving. A sparse maximum convolutional neural network was proposed to recognize difficult traffic signs through hierarchical classification with high accuracy [54]. Currently, the implementation of autonomous driving technology relies on the effective participation and control takeover of drivers. Therefore, even with autonomous driving technology, the perception of traffic signs and comfort on urban roads by drivers remains a pertinent research direction.

## Ethics declarations

All participants provided written informed consent to participate in the study and for their data to be published.

## Data availability statement

The data associated with this study has not been uploaded into a publicly available repository. However, data will be made available on reasonable request.

## CRediT authorship contribution statement

**Hui Hu:** Project administration, Funding acquisition, Formal analysis, Conceptualization. **Feng He:** Visualization, Supervision, Funding acquisition. **Yunwei Meng:** Writing – original draft, Visualization, Validation, Software, Methodology, Conceptualization. **Youzhang Yang:** Supervision, Resources, Investigation, Funding acquisition. **Binbin Li:** Writing – review & editing, Writing – original draft, Visualization, Validation, Project administration. **Guangyan Qing:** Writing – original draft, Methodology, Investigation.

## Declaration of competing interest

The authors declare that they have no known competing financial interests or personal relationships that could have appeared to influence the work reported in this paper.

## References

[1] Yang F., Yang J.J., Ma C.C., Wang S., Wang Z.G. (2021). Information threshold for guide signs at entrances and exits of ring expressway. China J. Highw. Transp..

[2] Du K.L., Du Z.G., Wang S.S., Xu F.Q., Mei J.L. (2023). Evaluation of traffic signs information volume in tunnel entrance area of low-grade highway. J. Wuhan Univ. Technol. (Transp. Sci. Eng.).

[3] Kang X., Kim W., Namgung M. (2020). Driver emotional and perceptual evaluation over various highway horizontal curves. KSCE J. Civ. Eng..

[4] Zang Y., Yan Z., Wu H., Gan P., Hu M., Wu W., Liu P., He G., Xiao J. (2023). An evaluation of the effect of urban tunnel lighting on driving comfort: a driving simulation study. Lecture Notes in Electrical Engineering.

[5] Meng Y.W., Wen L., Zhang X.Y., Qing G.Y. (2021). Research on dynamic evolution of driver's visual load based on catastrophe progression method in mountainous highway environment. IJIIMS.

[6] Sabek Bahaa, Jordan Srour F., El Mendelek Maria, Myriam El Khoury Malhame John Khoury (2024). Are you in the mood to pass? A study on the interplay of psychological traits and traffic on young drivers' overtaking behavior on two-lane, two-way highways. Transport. Res. Part F: Psychology and.

[7] Hu J. (2019). M.S. Thesis, Dept. Elect. Eng.

[8] Zheng L., Qiao X.Q., Ni T., Yang W., Li Y.N. (2021). Driver cognitive loads based on multi-dimensional information feature analysis. China J. Highw. Transp..

[9] Mohamad, D., Deros, B. M., Daruis, D. D. I., Ismail, A. R. Development of Regression Model Between Driving Comfort Perception and Muscle Contraction. Proceeding of 5th International Conference on Advances in Manufacturing and Materials Engineering. Lecture Notes in Mechanical Engineering. Springer, Singapore. 10.1007/978-981-19-9509-5_13. .

[10] Ju Y., Chen F., Li X., Lin D. (2023). Bibliometric study and critical individual literature review of driving behavior analysis methods based on brain imaging from 1993 to 2022. J. Traffic Transport. Eng..

[11] Richards L.G., Jacobson I.D., Kuhlthau A.R. (1978). What the passenger contributes to passenger comfort. Appl. Ergon..

[12] Wang Q. (2015).

[13] Xu J., Xiang Z.R., Zhi J.Y. (2020). Review on measurement and evaluation methods of visual comfort in lighting environment. Journal of Chongqing University of Technology (Natural Science).

[14] Min J.L., Cai M. (2020). Driver fatigue detection based on multi-scale wavelet log energy entropy of frontal EEG. China J. Highw. Transp..

[15] Meng Y.W., Cai H.Q., Qing G.Y. (2021). Preliminary quantitative research on characteristics of driving visual sensitive region in mountainous highway environment. IJIIMS.

[16] Lowden A., Anund A., Kecklund G. (2009). Wakefulness in young and elderly subjects driving at night in a car simulator. Accid. Anal. Prev..

[17] Tasaki M., Sakai M., Watanabe M., Wang H., Wei D. (2010). 2010 10th IEEE International Conference on Computer and Information Technology.

[18] Wang L.H., Li S.W., Zhou R.B., Yang Z.F., Ji B.K., Yao X.P. (2013). Impact of roadside landscape color on driver mean heart rate. J. Jilin Univ. (Eng. Technol. Ed.).

[19] Hu L.W., Yin X.F., Zhang S.H., Fan Z.J. (2020). Evaluation of driving comfort on slopes of secondary highway in mountainous area based on driver workload. Journal of Transportation Systems Engineering and Information Technology.

[20] Han L., Du Z., Wu K. (2023). Evaluation of traffic signs information volume at highway tunnel entrance zone based on driver's visual characteristics. Transport. Res. Rec..

[21] Liu K., Deng H. (2021). The relationship of the information quantity of urban roadside traffic signs and drivers' visibility based on information transmission. Int. J. Environ. Res. Publ. Health.

[22] Lyu N.C., Cao Y., Qin L., Wu C.Z. (2018). Research on the effectiveness of driving workload based on traffic sign information volume. China J. Highw. Transp..

[23] Yi X.B.W., Tang K.S., Li K.P. (2019). Location optimization of urban variable message sign based on amount of information. J. Tongji Univ. Nat. Sci..

[24] Hu L.W., Qi S.M., Sun Y.N., Li Y.P. (2014). Basic information analysis method of highway traffic signs. J. Kunming Univ. Sci. Technol. (Sci. Technol.).

[25] Luo C., Liu L., Zhang L. (2018). Traffic information measurement based on information theory. J. Southwest Jiaot. Univ..

[26] Lerner N.D. (2003).

[27] Lv Z., Wang H.X., Ding X. (2022). Effects of traffic signs on drivers' visual characteristics in grassland highway. Sci. Technol. Eng..

[28] Wang R.J., Zhou X.H. (2021). Review of cognition and evaluation of traffic signs on expressway. Traffic Information and Safety.

[29] Yu B., Chen Y. (2015).

[30] Karageorghis C.I., Kuan G., Mouchlianitis E., Payre W., Howard L.W., Reed N., Parkes A.M. (2022). Interactive effects of task load and music tempo on psychological, psychophysiological, and behavioural outcomes during simulated driving. Ergonomics.

[31] Chen F., Yang Y. (2019). Influence of tunnel entrance environment on driver's vision and physiology in mountainous expressway. IOP Conf. Ser. Earth Environ. Sci..

[32] Meng X., Chen X., Pan X. (2014). Research on traffic guidance system in underground parking lots based on visual driving adaptability. Cota International Conference of Transportation Professionals.

[33] Meng Y.W. (2017).

[34] Faure V., Lobjois R., Benguigui N. (2016). The effects of driving environment complexity and dual tasking on drivers' mental workload and eye blink behavior. Transport. Res. Part F: Psychology and Behaviour.

[35] Hong I., Iwasaki M., Furuichi T. (2006). Eye movement and driving behaviour in curved section passages of an urban motorway. Proc. Inst. Mech. Eng. - Part D J. Automob. Eng..

[36] Hankins T.C., Wilson G.F.A. (1998). Comparison of heart rate, eye activity, EEG and subjective measures of pilot mental workload during flight. Aviat Space Environ. Med..

[37] Yang Y.Q., Chen J.Y., Said M.E., Easa S. (2021). Internal causes of return trip effect based on eye movement and EEG indices. Transport. Res. F Traffic Psychol. Behav..

[38] Wu L. (2018).

[39] Kajiwara S. (2014). Evaluation of driver's mental workload by facial temperature and electrodermal activity under simulated driving conditions. Int. J. Automot. Technol..

[40] Jamil N.I., Rosle A.N., Ibrahim S.S., Baharuddin F.N. (2018). Parsimonious in factor identification using exploratory factor analysis. Adv. Sci. Lett..

[41] Hu L.W., Qi S.M., Sun Y.N. (2014). Basic information analysis method of highway traffic signs. J. Kunming Univ. Sci. Technol. (Sci. Technol.).

[42] Liu B.H., Sun L.S., Rong J. (2011). Based on eye movement parameters of the pilot symbol visual cognition behavior study. Journal of Transportation Systems Engineering and Information Technology.

[43] Liao X.F., Guan Z.J., Lu K. (2012). Evaluation index and standard of cognizability of Chinese traffic signs. Highways.

[44] Brookhuis K.A., Waard D. (2010). Monitoring Drivers' mental workload in driving simulators using physiological measures. Accid. Anal. Prev..

[45] Bella F. (2013). Driver Perception of roadside configurations on two-lane rural roads: effects on speed and lateral placement. Accid. Anal. Prev..

[46] Wang S., Du Z., Jiao F. (2020). Drivers' visual load at different time periods in entrance and exit zones of extra-long tunnel. Traffic Inj. Prev..

[47] Zheng H.R., Rasouli S., Du Z.G., Wang S.S. (2024). Association between length of upstream tunnels and visual load in connection zones of highway tunnel groups. Tunn. Undergr. Space Technol..

[48] Shang Y., Zhu S.L., Qi C.H. (2021). Long straight section of grassland best threshold information research. China Saf. Sci. J..

[49] Wang Y., Weidmann U.A., Wang H. (2017). Using catastrophe theory to describe railway system safety and discuss system risk concept. Saf. Sci..

[50] Castro S., Cooper J., Strayer D. (2016). Validating two assessment strategies for visual and cognitive load in a simulated driving task. Proc. Hum. Factors Ergon. Soc. Annu. Meet..

[51] Meng, Y. W., Quan, Z. Y., Wang, Z. X. Research on Driving Comfort on Highway Tunnel Portal Sections Based on Coordination of Luminance and Speed. Journal of Transportation Systems Engineering and Information Technology. Available: http://kns.cnki.net/kcms/detail/11.4520.U.20240626.1053.002.html. .

[52] Han L., Du Z., Wang S. (2022). Analysis of traffic signs information volume affecting driver's visual characteristics and driving safety. Int. J. Environ. Res. Publ. Health.

[53] Li Z., Fu R., Wang C. (2020). Effects of linear acceleration on passenger comfort during physical driving on an urban road. Int. J. Civ. Eng..

[54] Liu J., Ge H., Li J., He P., Hao Z., Hitch M. (2022). How can sustainable public transport be improved? A traffic sign recognition approach using convolutional neural networks. Energies.

